# Efeito de uma intervenção em aulas de educação física sobre a redução do comportamento sedentário em adolescentes

**DOI:** 10.1590/0102-311XPT211023

**Published:** 2024-12-20

**Authors:** Anísio Luis da Silva Brito, Rafael Miranda Tassitano, Edilânea Nunes Mélo, Simone José dos Santos, Jorge Mota, Mauro Virgílio Gomes de Barros

**Affiliations:** 1 Centro Universitário Maurício de Nassau, Caruaru, Brasil.; 2 - University of Illinois at Urbana Champaign, Urbana, U. S. A.; 3 Escola Técnica Estadual José Humberto de Moura Cavalcanti, Limoeiro, Brasil.; 4 Universidade de Pernambuco, Recife, Brasil.; 5 Universidade do Porto, Porto, Portugal.

**Keywords:** Comportamento Sedentário, Educação Física, Adolescente, Sedentary Behavior, Physical Education, Adolescent, Conducta Sedentaria, Educación Física, Adolescente

## Abstract

O objetivo foi analisar o efeito de diferentes estratégias de intervenção nas aulas de educação física sobre a redução do tempo em comportamento sedentário. Trata-se de uma intervenção randomizada, de base escolar e característica fatorial, realizada com estudantes de 1º ano do ensino médio de 11 escolas de tempo integral, em três situações de intervenção: (A) escolas com duas aulas extras de educação física por semana; (B) escolas com ações de treinamento e engajamento para professores de educação física; (C) escolas com a união das estratégias A e B; e (D) um grupo de escolas controle. Acelerometria foi utilizada para obter a medida de efeito do desfecho principal (comportamento sedentário). Regressão linear foi utilizada tanto para a análise da variação do tempo em comportamento sedentário entre o *baseline* e pós-intervenção como para a análise de potenciais fatores mediadores da relação entre exposição e desfecho. Participaram do estudo 1.295 estudantes. A intervenção B foi capaz de reduzir significativamente o tempo em comportamento sedentário de meninos (61,5 minutos, p < 0,01) e meninas (34,8 minutos, p = 0,04) em comparação ao grupo controle, demonstrando respectivamente tamanhos de efeitos médio (0,61) e pequeno (0,32). A redução do tempo em comportamento sedentário entre os estudantes do grupo B foi significativamente mediada pela redução da amotivação (AMO) (meninos c: -0,402; e meninas c: -0,376). A oferta de formação continuada para professores de educação física se mostrou estratégia fundamental para reduzir o tempo em comportamento sedentário dos adolescentes, sendo essa redução mediada pela diminuição da AMO para as aulas de educação física.

## Abstract (en)

This study aimed to analyze the effect of different intervention strategies in physical education classes on the reduction of sedentary time. This is a randomized, school-based intervention with factorial characteristics, carried out with freshmen high school students from 11 full-time schools, allocated in three intervention situations: (A) schools with two extra physical education classes per week; (B) schools with training and engagement actions for physical education teachers; (C) Schools with both A and B strategies; and (D) a group of control schools. Accelerometry was used to obtain the effect measure of the main outcome (sedentary behavior). Linear regression was used to analyze the time variation in sedentary behavior between baseline and post-intervention, as well as to analyze potential mediating factors of the relation between exposure and outcome. A total of 1,295 students participated in the study. Intervention B significantly reduced the time in sedentary behavior of boys (61.5 minutes, p < 0.01) and girls (34.8 minutes, p = 0.04) when compared to the control group, showing respectively medium (0.61) and small (0.32) effect size. The reduction in time in sedentary behavior among students in group B was significantly mediated by reduced lack of motivation (AMO) (boys c: -0.402, and girls c: -0.376). The offer of continuing training for physical education teachers proved to be a fundamental strategy to reduce the sedentary time of adolescents, which was mediated by the AMO reduction for physical education classes.

## Resumen

El objetivo fue analizar el efecto de diferentes estrategias de intervención en las clases de educación física en la reducción del tiempo de comportamiento sedentario. Se trata de una intervención escolar, aleatorizada y con características factoriales, realizada con alumnos de 1.º de secundaria de 11 colegios de jornada completa, asignados a 3 situaciones de intervención: (A) escuelas con dos clases extras de educación física por semana; (B) escuelas con acciones de formación y compromiso para profesores de educación física; (C) escuelas con la combinación de las estrategias A y B; y (D) un grupo de escuelas de control. Se utilizó acelerometría para obtener la medida del efecto del desenlace principal (comportamiento sedentario). Se utilizó la regresión lineal tanto para analizar la variación en el tiempo en el comportamiento sedentario entre el inicio y la posintervención, como para analizar los posibles factores mediadores de la relación entre la exposición y el desenlace. Participaron en el estudio 1.295 estudiantes. La intervención B logró reducir significativamente el tiempo dedicado a comportamiento sedentario en niños (61,5 minutos, p < 0,01) y niñas (34,8 minutos, p = 0,04) en comparación con el grupo control, lo que demuestra respectivamente tamaños de efecto mediano (0,61) y pequeño (0,32). La reducción del tiempo de comportamiento sedentario entre los estudiantes del grupo B fue significativamente mediana por la reducción de la desmotivación (AMO) (niños c: -0,402 y niñas c: -0 376). La oferta de formación continua para profesores de educación física resultó ser una estrategia fundamental para reducir el tiempo de conducta sedentaria entre los adolescentes, y esta reducción mediada por la disminución de la AMO para las clases de educación física.

## Introdução

Nos últimos anos, o corpo do conhecimento acerca do comportamento sedentário entre adolescentes tem crescido de forma exponencial. Estão bem documentados os efeitos negativos a curto e longo prazo do comportamento sedentário sobre desfechos de saúde física e mental entre os jovens ^1^ ^,^ ^2^ ^,^ ^3^. Em adição, a maioria dos estudos nacionais e internacionais tem relatado elevada prevalência de exposição a comportamento sedentário nesse subgrupo ^4^ ^,^ ^5^. Tais evidências são frequentemente utilizadas como justificativa da necessidade de se elaborar ações com foco na promoção de estilos de vida mais saudáveis entre jovens. Os estudos apontam para intervenções de caráter comunitário e de base escolar como efetivas para esse fim ^6^ ^,^ ^7^ ^,^ ^8^ ^,^ ^9^.

Revisões sistemáticas têm mostrado perspectivas animadoras com relação a resultados de intervenções escolares para a redução do comportamento sedentário entre adolescentes, indicando que tais intervenções devem ser encorajadas como estratégias primárias na redução desse comportamento, ainda que esses resultados sejam modestos e que algumas limitações devam ser consideradas, como: falta de ações específicas para se combater o comportamento sedentário; ausência de avaliação de processo; e ensaios com amostras não representativas ^7^ ^,^ ^9^ ^,^ ^10^. 

Diante disto, encontrar estratégias de intervenção adequadas, no contexto escolar, para reduzir o comportamento sedentário, assim como fornecer resultados consistentes dos seus efeitos a curto e a longo prazo, é um dos maiores desafios na área de saúde pública ^7^. Para isso, especialistas, visando dar maior qualidade às intervenções de promoção de estilos de vida saudáveis e redução do comportamento sedentário, têm sugerido algumas estratégias para obter as medidas de efeito, bem como ações nas aulas de educação física, sendo essas: (1) obtenção de informações referentes a outras formas de comportamento sedentário (tempo total sentado, tempo sentado em atividades de lazer com os amigos, tempo sentado na escola, tempo sentado no deslocamento, entre outros) e não apenas sobre o tempo de TV ou tempo de tela total ^9^ ^,^ ^11^ ^,^ ^12^; (2) utilização de sensores de movimento para mensuração do tempo em comportamento sedentário ^7^; (3) engajamento e formação continuada para professores de educação física ^13^; e (4) maior oferta de aulas de educação física por semana ^14^ ^,^ ^15^.

Logo, é necessário entender o efeito que essas ações podem exercer sobre a redução da exposição ao comportamento sedentário. Portanto, o objetivo deste estudo foi analisar o efeito de uma intervenção nas aulas de educação física sobre a redução do tempo de exposição ao comportamento sedentário em adolescentes.

## Métodos

### Delineamento

Este estudo é caracterizado como um ensaio comunitário randomizado de base escolar e de característica fatorial e faz parte do projeto *Saúde, Cognição e Desenvolvimento Escolar* (SACODE), que promove a intervenção para a redução do comportamento sedentário e melhoria da função cognitiva a partir das aulas de educação física. Em suma, o projeto teve como objetivo implementar estratégias de intervenção nas aulas de educação física que visavam à redução de exposição a indicadores do comportamento sedentário, melhoria de indicadores de cognição e desempenho escolar de estudantes do Ensino Médio.

### Intervenção

Este estudo foi conduzido em quatro grupos, sendo três expostos às estratégias de intervenção e um exposto a uma situação controle, distribuídos da seguinte forma: grupo intervenção A - aumento do número de aulas de educação física; grupo intervenção B - ações de treinamento e engajamento para os professores de educação física; grupo intervenção C - combinação das estratégias A e B; e grupo D - controle.

A intervenção realizada com o grupo intervenção A foi aumentar o número de aulas de educação física de duas para quatro aulas/semana. Cada escola organizou a sua grade curricular de modo a ofertar as aulas extras dentro do horário regular.

A intervenção conduzida no grupo intervenção B foi oferecer ações de treinamento e engajamento para os professores de educação física. Essa oferta foi realizada por meio de um curso de aperfeiçoamento organizado pelos pesquisadores responsáveis pelo projeto e certificado pela Universidade de Pernambuco (UPE), com carga horária total de 120 horas, sendo 30 delas divididas em seis momentos presenciais. A cada encontro, os professores recebiam material de apoio composto por textos relacionados à temática a ser trabalhada. As demais horas do curso foram destinadas à realização de um projeto de ação comunitária desenvolvido pelos professores de educação física em conjunto com os estudantes do primeiro ano do Ensino Médio de suas escolas. O curso tinha como objetivo abordar conteúdos relacionados aos principais desfechos de interesse do projeto SACODE, como: atividade física, comportamento sedentário, aptidão física, funcionamento do cérebro adolescente, saúde mental, relacionamentos e isolamento social e qualidade do sono. Além disso, esses encontros foram um ambiente fértil para o resgate de conteúdos de cunho didático-pedagógico que foram discutidos para apoiar os professores em seu trabalho docente, em que foram abordados a elaboração de objetivos, a seleção dos conteúdos, as estratégias metodológicas e o processo avaliativo nas aulas de educação física. 

### População e amostra

A população alvo do estudo foi composta por adolescentes matriculados em unidades públicas de ensino denominadas Escolas de Referência em Ensino Médio (EREM). Essas unidades estão localizadas na maioria dos municípios pernambucanos, sendo 173 funcionando em horário integral e 172 em jornada semi-integral. Por razões logísticas e operacionais definidas junto à Secretaria de Educação e Esporte de Pernambuco, o contexto de intervenção foi delimitado apenas à 8ª Gerência Regional de Educação Vale do Capibaribe (GREVC) cuja sede está situada no município de Limoeiro. Esta gerência é composta por 16 municípios e contém, pelo menos, uma EREM em cada um deles. Além disso, decidiu-se tornar elegíveis para o estudo apenas os alunos de primeiro ano devido à intenção de conduzir uma avaliação de acompanhamento (*follow-up*) aproximadamente um ano após o fim da intervenção.

As 11 EREM com jornada integral da GREVC foram consideradas elegíveis para compor o estudo na condição de intervenção ou de controle. Elas foram randomicamente selecionadas e avaliadas do início e ao final do ano letivo de 2017. Inicialmente, procedeu-se à classificação das escolas considerando os seguintes critérios: (1) espaços físicos para as aulas de educação física, cobertos ou descobertos; (2) número de professores de educação física; (3) número de turmas; e (4) média de estudantes por turma. Ao final desse processo foram obtidos três agrupamentos de escolas com características semelhantes entre si, sendo dois com quatro escolas e um com três escolas. Posteriormente, as escolas de cada agrupamento foram sorteadas para a condição controle e para cada uma das três condições de intervenção. A descrição desses procedimentos foi publicada em estudo anterior ^16^.

Nas 11 escolas, todos os estudantes matriculados na primeira série do Ensino Médio foram convidados a participar do estudo e, com aceite e autorização dos pais ou responsáveis legais, foram submetidos aos testes e questionários previstos no protocolo de investigação. Estimou-se, a priori, uma taxa de recusa entre 10% a 15%, bem como um percentual de perdas que poderia alcançar 50% da amostra inicial.

O tamanho amostral previsto, descontadas as previsões de recusas e abandonos, deveria estar em torno de 880 estudantes, sendo 220 em cada grupo do modelo fatorial. Considerando o alcance do tamanho amostral de 220 participantes por grupo, seria possível detectar uma diferença de risco/prevalência de 10% nas variáveis de desfecho dicotômicas entre o grupo controle e qualquer um dos esquemas de intervenção, considerando um nível de confiança e poder fixados em 95% e 80%, respectivamente.

No entanto, para medir o efeito da intervenção sobre o comportamento sedentário medido de forma direta por meio de acelerometria, apenas parte desses estudantes foram selecionados de forma aleatória usando a lista de matriculados, por turma. Tal decisão se justifica pelo tempo e número limitado de acelerômetros disponíveis para realização do monitoramento.

### Instrumentos de medida

### Comportamento sedentário

O comportamento sedentário foi medido por meio de acelerômetros triaxiais GT3X+ (ActiGraph; https://blog.theactigraph.com/news/actigraph-expands-headquarters-in-downtown-pensacola). O protocolo de utilização dos aparelhos consistiu em: (1) o adolescente utilizar o acelerômetro durante sete dias consecutivos; (2) utilizar o aparelho ao lado direito de sua cintura, fixado por uma cinta elástica; e (3) retirar o aparelho apenas para dormir, tomar banho, realizar atividades aquáticas e lutas. Apesar de reconhecer as limitações associadas ao uso de acelerômetros para medir o comportamento sedentário, é importante destacar que há, na literatura, indicação para o uso de tal instrumentação para estudos em ambiente escolar ^17^.

O software ActiLife (https://actigraphcorp.com/actilife/) foi utilizado para a programação e o download dos dados. Os aparelhos foram programados com *epoch* de 15 segundos, por ser um padrão recomendável para amostras na faixa etária do estudo ^18^. Para redução dos dados de acelerometria, foi utilizado o programa Propero (https://sourceforge.net/projects/propero/), adotando-se como válidos os períodos de monitoramento que atendessem os seguintes critérios: oito ou mais horas por dia de monitoramento, excluindo-se os períodos de 60 minutos consecutivos sem registro de aceleração (zeros consecutivos); três ou mais dias de monitoramento, sendo um deles do final de semana ^19^. 

Para a definição do c, foi utilizado o ponto de corte de < 25 contagens/15 segundos ^20^. A variável tempo diário em comportamento sedentário foi determinada pelo somatório do tempo de exposição total durante a semana (segunda a sexta-feira) multiplicado por cinco, e o tempo total em dias de final de semana (sábado e domingo) multiplicado por dois, sendo o resultado dividido por sete, obtendo-se a média ponderada do tempo em comportamento sedentário em minutos por dia.

O delta do tempo em comportamento sedentário foi criado para analisar a variação do tempo entre os pontos *baseline* e pós-intervenção. Essa variável foi determinada pela subtração da média em comportamento sedentário no pós-intervenção pelo do *baseline*.

### Demais variáveis

As demais variáveis do estudo, como as demográficas e mediadoras dos efeitos das intervenções testadas sobre a redução do tempo em comportamento sedentário, foram obtidas mediante aplicação de um questionário adaptado para este estudo. Esse questionário passou por uma testagem em estudo piloto a fim de determinar seus indicadores de reprodutibilidade. Ele foi aplicado em adolescentes da mesma faixa etária, matriculados em uma escola que não fazia parte da amostra, com um intervalo de sete dias entre as aplicações. Os valores dos indicadores de consistência de medidas de teste-reteste variaram de moderados a altos (concordância kappa = 0,7 a 1) para a maioria dos itens.

As variáveis demográficas do estudo foram sexo (masculino e feminino) e idade (anos). As variáveis mediadoras do estudo foram aquelas relacionadas à motivação para as aulas de educação física: (1) motivação externa (ME); (2) motivação intrínseca (MI); e (3) amotivação (AMO), sendo cada uma composta por três questões. Para a opção de resposta em cada uma das questões utilizou-se a escala Likert (1 - discordo plenamente; 2 - discordo bastante; 3 - discordo no geral; 4 - nem concordo e nem discordo; 5 - concordo no geral; 6 - concordo bastante; 7 - concordo plenamente).

### Análise dos dados

Os dados foram analisados mediante utilização do programa SPSS para Windows (https://www.ibm.com/). As análises descritivas das variáveis contínuas foram efetuadas a partir da determinação de médias e suas respectivas medidas de dispersão.

Para comparar as médias entre os grupos de alocação no *baseline*, optou-se por utilizar o teste de análise de variância (ANOVA) para um fator devido à distribuição normal dos dados. Já a análise da variação do tempo diário médio em comportamento sedentário entre o *baselin*e e o pós-intervenção foi realizada mediante utilização de regressão linear, no qual se observou quais das intervenções testadas apresentavam mudanças significativas (p < 0,05) nas estimativas de tempo em comportamento sedentário entre os pontos de avaliação.

Os tamanhos de efeito foram calculados a partir do desvio padrão (DP) e da diferença entre as médias do tempo em comportamento sedentário do *baseline* e pós-intervenção. Tais efeitos foram incluídos no estudo para demonstrar a magnitude das diferenças, podendo esses efeitos serem considerados pequenos (d = 0,2-0,3), moderados (d = 0,4-0,7) e grandes (d ≥ 0,8). Para as análises de mediação, todas as alterações das variáveis do modelo foram expressas pelos resíduos dos valores finais regredidos nos valores da linha de base.

Devido à hipótese de que meninos e meninas teriam respostas diferentes, as análises foram estratificadas por sexo. A análise de mediação de séries múltiplas foi realizada com base no modelo 6 proposto por Hayes, por meio do macro denominado PROCESS v3.1 para SPSS (https://processmacro.org/index.html), utilizando 5.000 amostras de *bootstrap*. Em suma, estimar o efeito total (c), o efeito direto (c’) e o efeito indireto (ind).

A variável independente do modelo (X) foi cada um dos três grupos de intervenção *versus* o grupo controle expressos por uma variável dicotômica (grupo controle D = 0 *versus* grupo de intervenção = 1). Mudanças no tempo em comportamento sedentário foi a variável dependente (Y) e as variáveis mediadoras foram as mudanças na ME (M1), na MI (M2) e AMO (M3). Um total de sete efeitos indiretos foram calculados com base em todas as combinações de caminhos entre as variáveis X ( Y (ind1, ind2, ind3, ind4, ind5, ind6 e ind7). Além disso, 21 efeitos indiretos específicos foram verificados (todas as combinações de um efeito indireto menos outro). O efeito indireto total é igual à soma de todos os efeitos indiretos específicos, já o efeito total (c) é igual ao efeito direto (c’) somado ao efeito indireto total.

### Considerações éticas

Todos os procedimentos aplicados durante a realização do projeto do qual este estudo faz parte foram avaliados e aprovados pelo Comitê de Ética em Pesquisa com Seres Humanos da UPE (protocolo CAAE: 55741016.0.0000.5207). O estudo está também registrado na Plataforma de Registro Brasileira de Ensaios Clínicos (registro nº RBR-88tgky).

## Resultados

Do total de alunos entrevistados no *baseline* (n = 1.296), uma subamostra de 854 adolescentes selecionados de forma aleatória, por meio da lista de matriculados, foram monitorados via acelerometria; no entanto, após redução dos dados, um total de 151 estudantes avaliados foram excluídos por não cumprirem os critérios mínimos para se considerar um monitoramento como válido. Com isso, a subamostra final de dados no *baseline* foi de 703 casos.

Entre os estudantes monitorados no *baseline*, 56,9% (n = 400 estudantes com dados válidos) eram do sexo feminino e a média de idade foi de 15 anos (DP = 1,1), com a inclusão de estudantes com idades entre 13 a 15 anos (Tabela 1).

**Tabela 1:** Características de estudantes de 1º ano com dados de acelerometria válidos na linha de base do projeto, estratificadas por grupo de alocação e sexo. Pernambuco, Brasil, 2017 (N = 12.195).

*Baseline*	Grupo intervenção A	Grupo intervenção B	Grupo intervenção C	Grupo controle D	Valor de p
Meninos					
Estudantes [n (%)]	88 (29,8)	87 (29,5)	60 (20,3)	60 (20,3)	-
Idade [ $\bar{X}$ (DP)]	15,2 (1,1)	14,8 (1,1)	15,3 (1,4)	15,3 (1,2)	< 0,01 *
Tempo em comportamento sedentário [ $\bar{X}$ minutos (DP)]	547,5 (86,7)	581,9 (89,9)	528,1 (107,6)	525,1 (54,5)	0,06
Motivação Intrínseca [ $\bar{X}$ (DP)]	13,8 (2,7)	13.7 (2,8)	14,1 (3,8)	13,3 (3,2)	0,54
Motivação Extrínseca [ $\bar{X}$ (DP)]	16,1 (3,7)	16,9 (2,7)	17,1 (3,9)	16,5 (3,9)	0,48
Amotivação [ $\bar{X}$ (DP)]	7,1 (3,5)	7,7 (2,9)	7,2 (4,7)	6,5 (4,0)	0,50
Meninas					
Estudantes [n (%)]	96 (24,0)	133 (33,3)	82 (20,5)	89 (22,3)	-
Idade [ $\bar{X}$ (DP)]	15,0 (1,3)	14,5 (0,8)	15,1 (0,7)	15,0 (0,9)	< 0,01 **
Tempo em comportamento sedentário [ $\bar{X}$ minutos (DP)]	578,7 (93,8)	576,4 (72,0)	565,7 (78,7)	565,1 (72,1)	0,54
Motivação Intrínseca [ $\bar{X}$ (DP)]	13,6 (2,7)	13,8 (2,7)	12,6 (3,5)	13,9 (2,3)	0,02 *
Motivação Extrínseca [ $\bar{X}$ (DP)]	15,9 (3,2)	16,2 (3,1)	14,8 (4,2)	16,2 (3,0)	0,04 *
Amotivação [ $\bar{X}$ (DP)]	7,4 (3,8)	6,5 (3,4)	7,5 (4,2)	7,8 (4,2)	0,16

DP: desvio padrão.

Grupo intervenção A: aumento do número de aulas de educação física; grupo intervenção B: ações de treinamento e engajamento para os professores de educação física; grupo intervenção C: combinação das estratégias A e B; grupo D: controle.

* *PostHoc* meninos: idade A *versus* B e B *versus* C;

** *PostHoc* meninas: idade A *versus* B, B *versus* C e B *versus* D; motivação intrínseca A *versus* B e A *versus* D; motivação externa C *versus* D.

No pós-intervenção, buscou-se avaliar os 854 adolescentes monitorados por acelerômetro no *baseline*, mas 155 estudantes não foram encontrados na escola no momento da coleta de dados ou se recusaram a usar o aparelho. Além disso, as limitações de tempo e de equipamentos fizeram com que não fosse possível o monitoramento via acelerometria em estudantes de duas escolas, sendo perdidos 162 monitoramentos. Assim, apenas 537 dos 854 monitoramentos realizados no *baseline* puderam ser repetidos seis meses depois.

**Figura 1:**
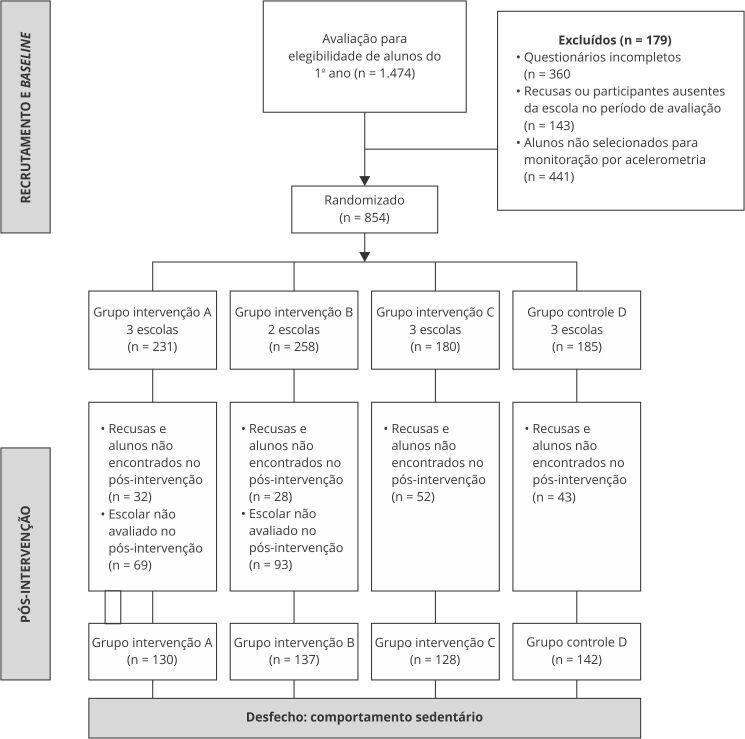
Fluxograma do projeto e tamanho da amostra para medida de acelerometria, estratificado por grupo de alocação.

Após a redução dos dados, ainda foram excluídos mais 146 casos por não cumprirem os critérios mínimos para se considerar um monitoramento válido, totalizando uma subamostra final de 391 casos no pós-intervenção (Figura 1). Do total de estudantes monitorados por meio de acelerometria no pós-intervenção, 56,1% (n = 216 estudantes com dados válidos) eram do sexo feminino, com idade média de 15 anos (DP = 1,0).

Com relação à variação do tempo médio em comportamento sedentário entre o *baseline* e o pós-intervenção, pode-se observar que apenas o grupo intervenção B produziu efeito na redução do tempo médio em comportamento sedentário dos estudantes. Entre meninos e meninas, essa redução foi, respectivamente, de 61,5 minutos (intervalo de 95% de confiança − IC95%: -102,5; -20,6; p < 0,004) e de 34,8 (IC95%: -67,3; -2,4; p < 0,035), quando comparados aos seus pares alocados no grupo controle. Entre os meninos, essa redução representa um efeito moderado (d de Cohen = 0,61) do grupo intervenção B sobre a redução do tempo diário médio em comportamento sedentário; já para as meninas, o efeito foi mais modesto (d de Cohen = 0,32), portanto, considerado pequeno.

Na análise de regressão linear múltipla, foi observado que a relação entre o grupo intervenção B e a redução do tempo médio em comportamento sedentário nos estudantes foi mediada de forma significativa pela diminuição da AMO, mas não pela ME e MI, tanto para os meninos como meninas (Tabela 2). Para os meninos, o efeito direto da intervenção B sobre a diminuição do tempo em comportamento sedentário foi de -0,424 (IC95%: -0,789; -0,051; p = 0,02), já o efeito indireto considerando todos os mediadores foi de 0,022 (IC95%: 0,010; 0,028). Com isso, observa-se que o efeito total da intervenção foi de -0,402 (IC95%: -0,769; -0,041; p = 0,02) (Tabela 3). O modelo de análise adotado foi responsável por explicar 21% (F = 4,87; p = 0,02) da diminuição do tempo médio em comportamento sedentário no pós-intervenção, em que parte dessa redução (5%) pode ser atribuída aos mediadores incluídos no modelo (ME, MI e AMO). 

**Tabela 2:** Análise de múltiplos mediadores do grupo intervenção B * sobre a mudança do comportamento sedentário entre estudantes. Pernambuco, Brasil, 2017.

				Efeitos				
	a1	a2	a3	b1	b2	b3	c’	Indireto
Meninos	0,108	0,032	-0,468 **	-0,120	-0,138	-0,190 **	-0,424 **	0,022 **
Meninas	-0,071	0,146	-0,302 **	-0,301	0,112	-0,324 **	-0,432 **	0,056 **

Nota: a1: efeito da intervenção sobre a motivação externa; a2: efeito da intervenção sobre amotivação interna; a3: efeito da intervenção sobre a motivação; b1: efeito da motivação externa sobre o tempo em comportamento sedentário; b2: efeito da motivação interna sobre o tempo em comportamento sedentário; b3: efeito da amotivação sobre o tempo em comportamento sedentário; c’: efeito direto da intervenção sobre o tempo em comportamento sedentário.

Indireto: efeito indireto da intervenção sobre o tempo em comportamento sedentário, considerando todos os mediadores (motivação externa, motivação interna e amotivação).

* Grupo intervenção B: ações de treinamento e engajamento de professores de educação física.

** p < 0.05.

**Tabela 3:** Ajuste do modelo, efeito total, efeito direto e efeito indireto. Pernambuco, Brasil, 2017.

Grupo intervenção *	Ajuste do modelo			Efeito total: efeito direto + efeito indireto	Valor de p	Efeito direto: c’	Valor de p	Efeito indireto	Caminho indireto	C: ind menos 1	Efeito total da série de mediadores (%)
	R^2^ (%)	F	Valor de p								
Meninos											
A	5	0,245	0,63	-0,106 (-0,610; 0,540)	0,53	-0,066 (-0,410; 0,270)	0.70	NS	NS	NS	-
B	21	4,87	0,02	-0,402 (-0,769; -0,041)	0,02	-,424 (-0,789; -0,051)	0,02	0,022 (0,010; 0,028)	Ind3: 0,019 (0,008; 0,147)	NS	5
C	6	0,498	0,48	-0,127 (-0,489; 0,235)	0,48	-0,124 (-0,490; 0,235)	0,55	NS	NS	NS	-
Meninas											
A	12	2,12	0,14	0,215 (-0,071 -0,507)	0,14	0,256 (-0,047; 0,560)	0,09	NS	NS	C2: -0,153 (-0,308; -0,090)	-
B	22	7,66	< 0,001	-0,376 (-0,700; -0,050)	< 0,001	-0,432 (-0,748; -0,124)	< 0,001	0,056 (0,040; 0,074)	Ind3: 0,041 (0,030; 0,108)	NS	12,9
C	13	2,77	0,09	-0,265 (-0,580; -0,049)	0,09	-0,277 (-0,603; 0,040)	0,09	NS	NS	NS	-

c’: efeito direto do grupo intervenção B sobre o tempo em comportamento sedentário; F: significância global na análise de regressão; NS: não significativa; R²: coeficiente de determinação do modelo.

Nota: Ind3 = X ( M3 ( Y; X = grupo; Y = mudança no tempo em comportamento sedentário; M3 = mudança na amotivação; C2 = ind2 menos ind3.

* D: grupo controle que foi o grupo utilizado como referência para comparar os demais grupos que sofreram alguma intervenção.

Para as meninas, foram encontrados resultados na mesma direção, em que o efeito direto do grupo intervenção B sob a diminuição do tempo em comportamento sedentário foi de -0,432 (IC95%: -0,748; -0,124; p < 0,001); já o efeito indireto considerando as três variáveis mediadoras foi de 0,056 (IC95%: 0,040; 0,074). Com relação ao efeito total, foi identificado um valor de -0,376 (IC95%: -0,700; -0,05; p = 0,02) (Tabela 3). Nas meninas, o modelo final de análise foi responsável por explicar 22% (F = 7,66; p < 0.001) da redução do tempo diário médio em comportamento sedentário, sendo parte dessa diminuição (12,9%) explicada pela série dos mediadores ME, MI e AMO.

Os grupos intervenções A e C não apresentaram efeitos significativos sobre a redução do tempo diário em comportamento sedentário dos adolescentes participantes do estudo. No entanto, embora esses efeitos não tenham sido significativos, eles seguiram o mesmo sentido do grupo intervenção B entre os meninos.

Já entre as meninas, chama a atenção que o grupo intervenção A tenha seguido uma direção oposta, sugerindo um aumento do tempo diário em comportamento sedentário, embora não significativo (efeito total = 0,215; IC95%: -0,071; 0,507; p = 0,14). Ainda vale ressaltar que, entre as meninas, o grupo intervenção A foi responsável por aumentar a AMO e diminuir o nível de MI (C2: -0,153; IC95%: -0,308; -0,090).

## Discussão

Este estudo apresenta a análise do efeito de uma intervenção de base escolar com foco no aumento do número de aulas e no treinamento e engajamento de professores de educação física sobre a redução do tempo em comportamento sedentário em estudantes do Ensino Médio. Nele, foi possível observar que a intervenção B foi uma importante estratégia para atenuar o tempo em comportamento sedentário tanto em meninos como em meninas, e que esse efeito foi mediado por uma diminuição dos níveis de AMO, em ambos os sexos, para aulas de educação física.

Os resultados demonstram que, em escolas nas quais os professores de educação física participaram de ações de treinamento e engajamento, os rapazes tiveram, logo após o fim da intervenção, uma diminuição significativa de cerca de 61,5 minutos no seu tempo diário em comportamento sedentário; já para as moças, essa diminuição foi mais discreta, cerca de 34,8 minutos. 

Os achados apontados fazem emergir a ideia de que a estratégia de intervenção utilizada, mesmo simples e de baixo custo, foi capaz de produzir resultados semelhantes aos de outros estudos disponíveis na literatura, como no caso do estudo delineado por Brittin et al. ^21^, que testou se jovens expostos a novos ambientes escolares construídos (salas de aula ao ar livre, jardins, trilhas naturais, ginásios, dois grandes campos esportivos, salas de aulas com móveis dinâmicos e cadeiras com ajustes de altura com balanços) demonstrariam mudanças em seus padrões de comportamento sedentário e de atividade física. Os resultados encontrados por eles sugerem que essas modificações testadas na intervenção foram capazes de atenuar em aproximadamente 81 minutos/dia a exposição do tempo total em comportamento sedentário dos sujeitos.

No mesmo sentido, um estudo de síntese avaliou a eficácia das intervenções escolares destinadas a reduzir o tempo sedentário medido de forma objetiva em jovens. De 11 intervenções avaliadas, apenas cinco apresentaram redução significativa no tempo sedentário, demonstrando uma variação de diminuição de 45 minutos/dia a 60 minutos/dia no tempo total de exposição ao comportamento sedentário ^22^.

As diminuições no tempo em comportamento sedentário entre o *baseline* e o período pós-intervenção resultaram em tamanhos de efeito moderado (d = 0,61) para os rapazes e pequenos (d = 0,32) para as moças. Em uma metanálise, Friedrich et al. ^7^ analisaram os efeitos das estratégias dos programas de intervenção sobre o tempo de tela (assistir à televisão, jogar videogame e usar computador) em escolares e identificaram que houve efeito significativo na redução do tempo de tela, apresentando diferença nas médias padronizadas de -0,25 (p < 0,01), o que parece ser um efeito menor do que os obtidos neste estudo.

Essa diferença observada pode ser atribuída ao fato de que a maioria das intervenções analisadas tinham a redução do tempo em comportamento sedentário como desfecho secundário, produto do aumento primário dos níveis de atividade física e da melhoria dos padrões alimentares. Diferente disso, a identificação de que o comportamento sedentário tem uma forte relação com a modulação dos diferentes desfechos de interesse (cognição, desempenho escolar e isolamento social) do projeto SACODE é que o comportamento sedentário foi frequentemente abordado durante os treinamentos dos professores e sua redução foi o objetivo principal deste estudo. 

Além disso, a análise exclusiva do tempo de tela pode ter subestimado o tamanho do efeito encontrado na metanálise realizada por Friedrich et al. ^7^, pois, embora o tempo de tela represente as atividades sedentárias mais frequentemente realizadas por jovens, elas não representam toda a magnitude do problema. Portanto, o monitoramento por meio de acelerometria realizado neste estudo tem permitido ampliar a visão do tempo diário de exposição ao comportamento sedentário.

A heterogeneidade nos resultados acabam dificultando a comparação dos achados encontrados com os disponíveis na literatura, e podem ser considerados reflexo da diversificação na duração das intervenções, no período de acompanhamento, no tamanho amostral, na diversidade de modelos teóricos utilizados no processo de planejamento e delineamento das intervenções, na variedade de critérios utilizados para considerar monitoramentos como válidos nas avaliações baseadas em acelerometria e, especialmente, pelo elevado número de estratégias testadas entre os estudos que vão de único componente a múltiplos componentes ^7^ ^,^ ^22^ ^,^ ^23^ ^,^ ^24^.

A redução do comportamento sedentário apontada por este estudo foi modesta, mas pode ser considerada um passo fundamental para mudanças no padrão de comportamento desses jovens e, consequentemente, na sua saúde. Fortalecendo essa ideia, Penning et al. ^25^ demonstram que a redução do tempo sentado tem impacto positivo na melhoria do colesterol total, HDL e função cognitiva de adolescentes, equivalente a ganhos que representam seis meses na capacidade de atenção. 

Os resultados deste estudo sugerem, ainda, que os efeitos do grupo intervenção B sobre a redução do tempo diário de exposição ao comportamento sedentário de meninos e meninas perpassaram em partes por mudanças que a própria intervenção provocou nos níveis de amotivação para as aulas de educação física, apontando, assim, que a redução dos níveis de amotivação se configurou em aspecto mediador da relação entre intervenção e desfecho. 

Vale ressaltar que as demais intervenções, embora não tenham apresentado efeitos significativos sobre a redução do tempo em comportamento sedentário, seguiram a mesma direção dos achados aqui apresentados. No entanto, para as meninas, a intervenção A parece ter comportamento oposto ao seguido pelas demais, apresentando um aumento no tempo em comportamento sedentário, sendo perpassado pelo aumento da amotivação e pela diminuição da motivação intrínseca. Isso pode ter acontecido porque aumentar o número de aulas de educação física por semana sem oferecer condições aos professores para ressignificarem sua prática docente pode refletir em uma diminuição na motivação para essas aulas em um grupo já desmotivado; diminuindo, então, o significado de importância que o aprendizado oferecido pelas aulas tem para esse grupo.

Diante do exposto, as ações de treinamento e engajamento ofertadas aos professores de educação física neste estudo parecem ter exercido papel importante na redução do tempo em comportamento sedentário dos adolescentes. Isso pode ter acontecido pelo fato dessas ações terem se configurado em ambiente importante para a apropriação de novos saberes, de ressignificação e aprimoramento da prática docente por meio de uma troca mútua de experiências e conhecimentos entre os envolvidos, o que pode ter refletido em maiores níveis de motivação dos professores para a condução de sua rotina pedagógica, como aponta a literatura disponível ^26^ ^,^ ^27^. 

O ganho de motivação docente pode refletir na escolha de estilos de ensino mais motivadores e que identifiquem as reais necessidades dos alunos, incentivando a autonomia e ofertando maiores oportunidades de escolha e apoio à cooperação, que são aspectos fundamentais para o aumento dos níveis de motivação de estudantes para a apropriação de seus saberes escolares nas aulas de educação física ^28^.

A esse respeito, Ferrer-Caja & Weiss ^29^ afirmam que, quando estudantes percebem que a sua participação nas aulas de educação física promoveu aprendizado real, eles se concentram mais nas atividades, refletindo em maior esforço para buscar melhores desempenhos, o que faz emergir a ideia de que estudantes mais motivados apresentam resultados superiores quando comparados aos demais com menores índices de motivação. Esses resultados podem ser produto tanto de fatores internos (competência percebida, autonomia percebida, aparência física e objetivo) como das ações conduzidas pelos professores de educação física na sua prática docente e dos conteúdos por eles ministrados ^29^ ^,^ ^30^, reafirmando, portanto, a importância da formação de professores dessa disciplina como peça fundamental na condução de mudanças nos estilos de vida de jovens.

Este estudo apresenta achados promissores de intervenções nas aulas de educação física para a redução do tempo em comportamento sedentário de estudantes de Ensino Médio de escolas em tempo integral, demonstrando que ações simples, como a oferta de formação continuada para professores de educação física, podem se configurar como intervenções relevantes e de baixo custo para reduções significativas no padrão de comportamento sedentário de adolescentes, sendo ela mediada pela diminuição da amotivação para as aulas de educação física. 

Além disso, foi observado que o simples aumento no número de aulas de educação física, além de não apresentar efeito na redução do comportamento sedentário entre os estudantes, também repercutiu em aumentos dos níveis de amotivação e diminuição dos níveis de motivação intrínseca entre as meninas.

Embora sejam achados importantes, é necessário considerar algumas limitações durante a interpretação dos resultados. O uso de sensores de movimento pode levar a subestimativas do tempo em comportamento sedentário por não diferenciar com precisão as posições corporais (em pé ou sentado). Além disso, há diferentes critérios sugeridos na literatura para a redução dos dados, assim como propostas diversas em relação aos pontos de corte para caracterizar o comportamento sedentário. As perdas de mais de 55% dos casos, decorrentes da falta de monitoramento por acelerometria dos adolescentes na avaliação pós-intervenção e da exclusão de dados não válidos, constituem também uma fragilidade que deve ser considerada. É plausível supor que as perdas tenham impactado os resultados do estudo e sejam, também, uma possível explicação para o fato de que somente o grupo intervenção B (engajamento e treinamento) tenha produzido efeito na redução de comportamento sedentário. A ausência de informações sobre a variação do tempo em comportamento sedentário no *follow-up* impossibilita, também, identificar se o efeito da intervenção teria longo prazo.

Apesar dessas limitações, este estudo possui vários pontos fortes: (1) ser um estudo que avalia o impacto de uma intervenção de base escolar; (2) ser um estudo que acontece no ambiente natural, sem influência do pesquisador no controle do que os professores realizavam em suas aulas, fazendo com que essa experiência possa ser mantida na escola sem aumento dos gastos e desenvolvida em outras realidades escolares; (3) testar o impacto de aulas extras de educação física no currículo de escolas de ensino médio em tempo integral, que tendem a crescer no Brasil para atender às exigências do novo ensino médio; (4) contar com o apoio dos gestores e o envolvimento dos professores ao longo de todas as etapas do projeto; (5) abranger todas as escolas em tempo integral da GREVC existentes durante a realização da intervenção. É importante destacar que, embora a intervenção realizada tenha potencial para modificar outros comportamentos relacionados à saúde (e.g., atividade física), este estudo avaliou somente seu impacto no comportamento sedentário.

Com isso, se faz necessária a continuidade e a concentração de esforços da comunidade científica em busca de resultados cada vez mais robustos e consistentes sobre o papel da educação física escolar na adoção de estilos de vida mais saudáveis entre jovens. Nesse sentido, é fundamental que a proposição das novas intervenções a serem conduzidas considerem incluir nas análises informações sobre as medidas de efeitos a longo prazo (*follow-up*), buscar por estratégias mais efetivas para minimizar o número de perdas no seguimento. Além disso, é preciso agregar o indispensável protagonismo docente, incluindo-o como agente fundamental do processo de mudança no estilo de vida dos alunos, utilizando a experiência e as necessidades docentes em um planejamento horizontal das ações a serem conduzidas nos futuros estudos. Reforça-se que as perdas de participantes durante a intervenção podem ter influenciado alguns resultados e, por isso, a aplicação dos achados deve ser efetuada com cautela.
